# Development and feasibility of an oral health e-learning program for long-term Japanese overseas workers: a pilot randomized controlled trial

**DOI:** 10.1186/s12903-023-03361-9

**Published:** 2023-09-05

**Authors:** Kiriko Sasayama, Yasuko Momoi, Stuart Gilmour, Erika Ota

**Affiliations:** 1 Institute for Global Health Policy Research, Bureau of International Health Cooperation, National Center for Global Health and Medicine, 1-21-1 Toyama Shinjuku-ku, Tokyo, 162-8655 Japan; 2 Department of Operative Dentistry, Tsurumi University School of Dental Medicine, Yokohama, Japan; 3 Department of Public Health, St. Luke’s International University Graduate School of Public Health, Tokyo, Japan; 4 Department of Global Health Nursing, St. Luke’s International University Graduate School of Nursing, Tokyo, Japan; 5 Tokyo Foundation for Policy Research, Tokyo, Japan

**Keywords:** Overseas worker, Information-motivation-behavioural skills, Oral health, Self-care, E-learning program, Randomized controlled trial

## Abstract

**Background:**

This study aimed to assess the feasibility and acceptability of an oral health self-care e-learning intervention for overseas workers as well as the research procedures for a future controlled trial.

**Methods:**

We randomly allocated participants to either the intervention (n = 48) or control (n = 51) group. The intervention group received a standardized leaflet plus a theory-based oral health e-learning program. The control group received only the standardized leaflet. We assessed health behaviour related to fluoride toothpaste use, oral care knowledge, motivation, oral care self-efficacy, and oral health related quality of life (QoL). Chi-square and t test analyses were performed to make comparisons between the two groups. To evaluate the research process, participants in the intervention group were asked open-ended questions to assess the acceptability and feasibility of the research procedures in practice.

**Results:**

A total of 82 participants (Intervention = 36, Control = 46) were included in the analysis. The dropout rate was 17.2%. The modal time taken to complete the e-learning intervention was more than 30 min (33.3%). Of the 36 respondents in the intervention group, 27 (70.4%) said that the e-learning intervention had changed their behaviour. At the three months follow-up, oral care knowledge alone was improved in the e-learning group.

**Conclusion:**

This pilot study provides evidence that the theory-based self-care for oral health e-learning intervention is feasible in overseas workers. Next, this feasible and acceptable pilot study should be used with an appropriate sample size in a randomized controlled trial.

**Trial registration:**

The trial protocol was registered with UMIN-CTR (ID: UMIN000045883) on 27/10/2021.

## Background

Pretravel consultations are a major opportunity to educate travellers about health risks at their destination and how to mitigate them [1]. Such consultations provide information on basic health habits while traveling, such as frequent hand washing, seat belt use, safe sexual behaviour, and general issues such as injury and sun protection. The main topic of pretravel counselling is education about infectious diseases, with less focus on oral health. Many clinicians think of infectious diseases first as health problems for travellers, while oral health is a neglected issue [2, 3]. However, travellers often face various oral health problems while traveling and may need to interrupt or cut short their trip due to a lack of knowledge on how to prevent and manage minor dental problems, as well as limited access to dental care before departure [4].

One group particularly at risk of health problems during travel are voluntary service overseas (VSO) workers, who spend long periods of time working in remote or challenging environments in low-and middle-income countries. United Kingdom’s VSO was founded in 1958 and has been implemented in over 70 developing countries [5]. The third most common disease among individuals engaging in VSO is dental caries, and based on interviews on diseases and injuries among Japanese workers in developing countries, the most common disease among overseas volunteers is dental disease [6]. Therefore, interventions aimed at helping overseas workers learn self-care oral health activities prior to travel should be encouraged.

The aims of self-care for oral health activity are to achieve oral health-related goals through increased oral care knowledge and changes in attitude and behaviour. Interventions to improve oral health-related behaviours need to be designed based on a psychological model or theory [7]. The information-motivation-behavioural skills (IMB) model is a suitable for implementing behavioural change interventions [8] and has been applied to a range of health interventions including HIV/AIDS, alcohol consumption, type 2 diabetes, coronary artery disease, and endometrial cancer [9–13]. The model identifies information as “the initial prerequisite for performing health behaviours” [14], two factors of motivation governing beliefs about a particular health behaviour [12, 15] and perceived social support [15], and dimensions of behavioural skills and self-efficacy required to implement behavioural change [15].

In this study, we developed an evidence-based self-care for oral health e-learning program for Japanese overseas workers using the IMB model, and we assessed the feasibility and acceptability of a self-care for oral health e-learning intervention for long-term Japanese overseas workers.

## Methods

The study consisted of the development of an evidence-based self-care for oral health e-learning program (stage 1) and the measurement of the feasibility of this program (stage 2).

### Stage 1: development implications

The purpose of the program was to help overseas workers learn about oral problems, their possible causes, and prevention and management through e-learning. This program focused on educating people to learn about the latest evidence-based prevention methods and management for dental caries in addition to periodontal disease and other oral discomfort. Self-care was the focus of the program so that workers could maintain their oral health independently in low- and middle-income countries with limited access to healthcare and medical supplies.

#### Structure of the e-learning program

The program focused on typical adult dental diseases. It included oral care knowledge, self-care check procedures, prevention methods based on the latest evidence, videos about toothpaste use and flossing, information on oral illnesses and oral care products for long-term overseas travel, and finally, a comprehension quiz. The program provided informed evidence in an interactive, interesting, and easy-to-understand way using cartoons, videos, and interactive quizzes. The e-learning operation was outsourced to the cloud-based e-learning system “EDEN LMS” (https://eden.ac/e-learning/).

#### Elements of e-learning programs

The program aimed to help participants to (a) understand that dental disease is one of the most common diseases while staying abroad; (b) recognize common oral diseases (Pathophysiology and prevention of dental caries, periodontal disease, tooth grinding, tooth loss, and halitosis) in people in their 30 and 40 s and the need to prevent them now; (c) understand that the use of highly concentrated fluoride toothpaste can help prevent dental caries effectively; (d) execute proper brushing and flossing using highly concentrated fluoride toothpaste; and (e) determine which oral care products are needed before staying abroad.

#### Contents of the IMB model-based e-learning program

**Fig. 1:**
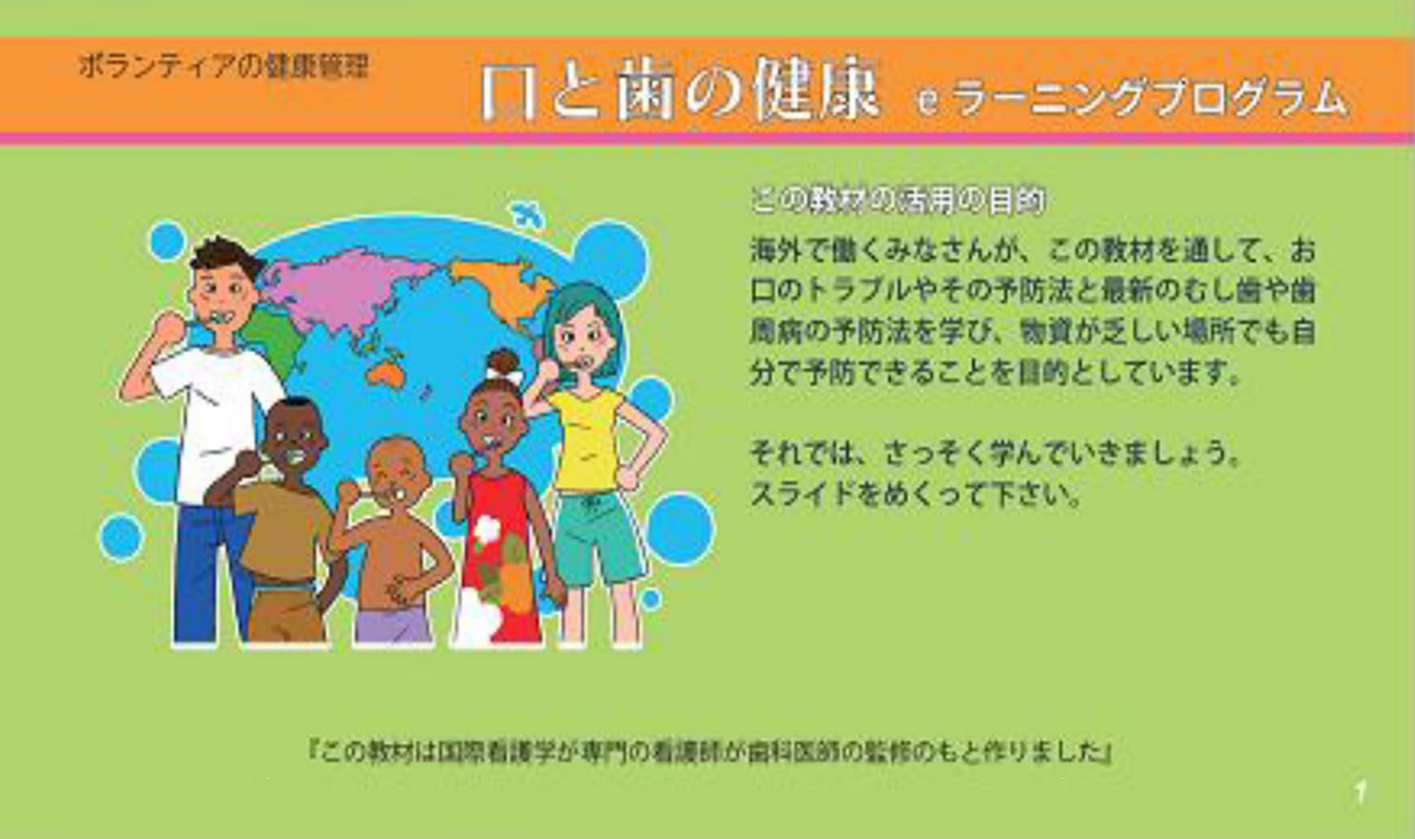
E-learning Content

**Fig. 2:**
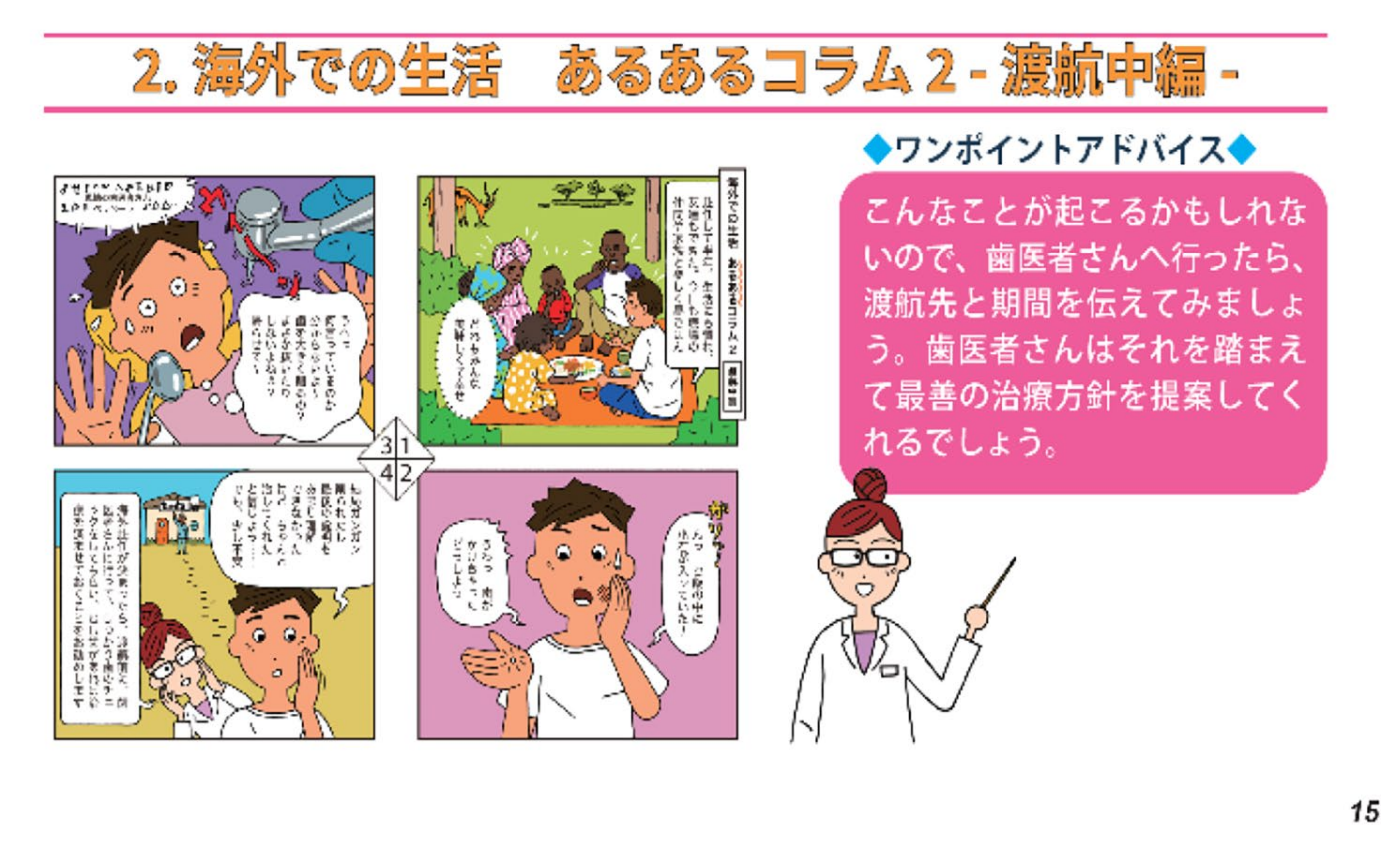
E-learning Content

The e-learning program comprised five chapters. The first chapter describes how the material can support life abroad and explains in simple terms what risks are present while staying overseas (Fig. 1). Cartoons were developed based on interviews with people who have stayed abroad, and used to explain common oral problems and how to prevent them (Fig. 2). The second chapter provides a self-check list to determine how many risk factors individuals have and what preventive measures can be implemented on a personal level.

**Fig. 3:**
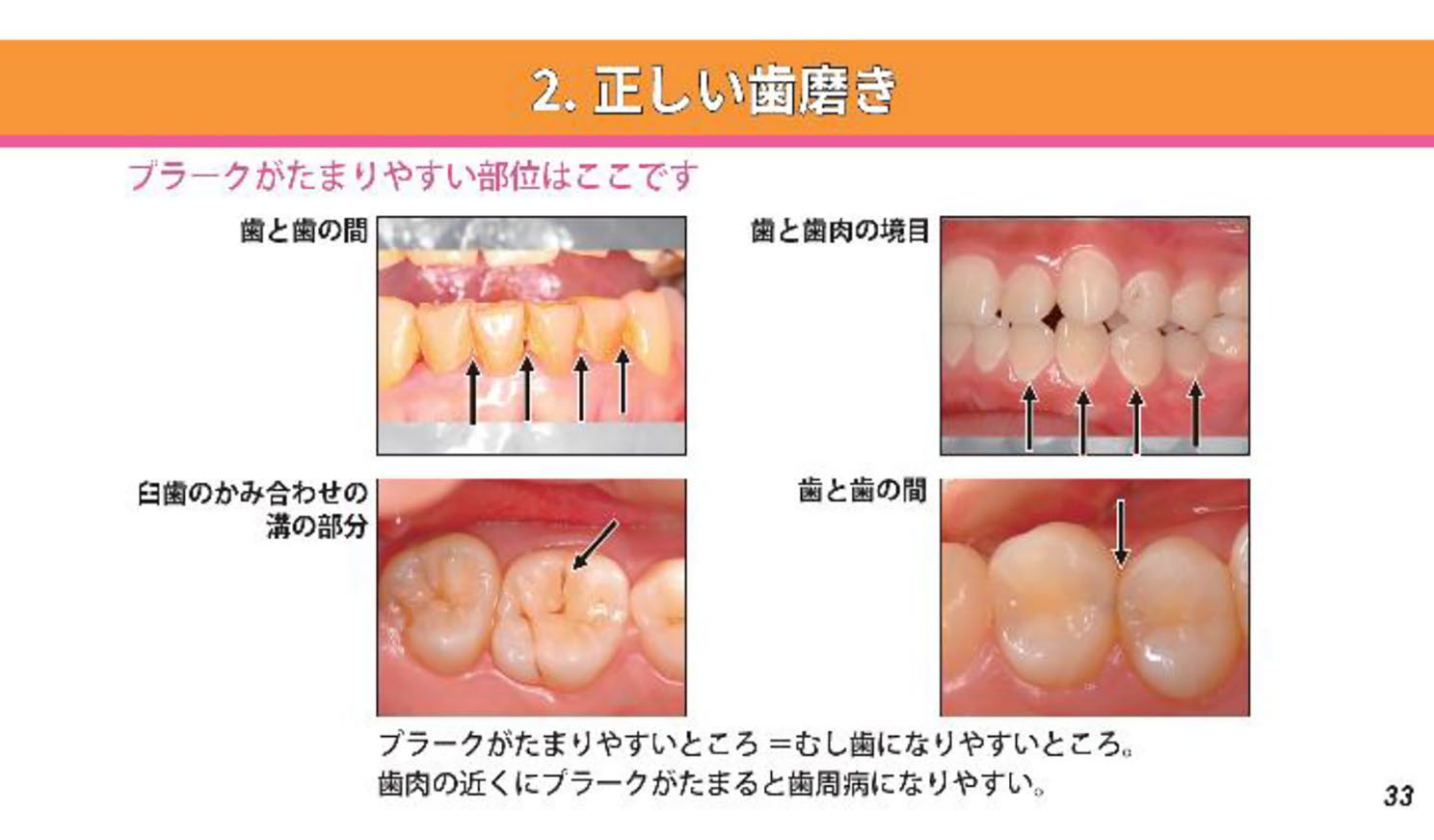
E-learning Content

The third chapter is the most important, as it explains the origins of oral problems and their care in a more practical way (Fig. 3). After users have gained some preliminary knowledge about dentistry in the third chapter, in the fourth chapter, they can comprehend what oral care they need to undergo before traveling abroad. In the final chapter, there is a test to check users’ understanding.

### Stage 2: feasibility

#### Study design and data collection

This pilot study was a parallel randomized controlled trial with two groups. Leaflets on self-care for oral health, that are commonly available in Japanese dental offices, were distributed to both groups. The intervention group was offered a leaflet plus a theory-based oral health e-learning program, while the control group received only the leaflet. Both groups were followed for 3 months. Block randomization to the intervention or control group was performed in a 1:1 ratio [16], stratified by gender, smoking history, and experience of having worked as a medical professional in the past. Randomization was carried out using a web-based randomization system. Each participant received either a leaflet and e-learning or only a leaflet, which was one of two codes. This allocation process was carried out by a research assistant who was not informed of the codes. The research participant codes and intervention codes were recorded in a chart for reference.

Participants were recruited from two overseas cooperation volunteer centres for volunteers dispatched by the Japanese government. Inclusion criteria were an interest in participating in the study, age between 20 and 44 years, plans to stay abroad for at least three months, ability to understand Japanese and no ongoing treatment for a chronic disease. Based on data showing that periodontal disease increases and people lose more teeth from the age of 40 years, the target group was set at 20–45 with the aim of promoting oral care at an earlier stage in the life cycle. Individuals who had dental pain or bleeding or who provided incomplete responses to the questionnaires were excluded from the research. Assessments were conducted at baseline and three months after intervention by questionnaires sent via e-mail. In addition, a descriptive design was adopted using a questionnaire to understand the acceptability of the intervention materials at the three-month follow-up in the intervention group.

#### Intervention and control

Leaflets on self-care for oral health were distributed to both groups. This leaflet was 35 pages long and covered periodontal disease, dental caries, dental hygiene, oral care, eating habits, and lifestyle. The leaflet was suitable for all ages and was available at general dental clinics. Before leaving for their assignment location, the intervention group was provided the URL code and passwords via e-mail to connect to the oral health e-learning program.

#### Sample size

We invited all volunteers who would be sent during the year, which is considered an eligibility criterion, to participate in this study. Data from the Volunteer Center confirmed that approximately 20 to 30 eligible individuals could participate on five occasions during the year after COVID-19 infection precautions had settled. Five recruitments per year would allow approximately 120 or more people to be invited to study in a year, thus 100 people were targeted.

#### Outcomes

Feasibility of trial design: feasibility of recruitment, randomization and follow-up of participants, and investigation of whether this evidence-based self-care for oral health e-learning program would be accepted by participants.

Intervention acceptability: Intervention completion times, user ratings of intervention quality and acceptability.

Preliminary evidence of effects: The three components of the IMB model (information, motivation and behavioural skills), oral health behaviour, and the oral health-related QoL resulting from those health behaviours were measured (see Measures).

## Measures

### Information

#### Oral care knowledge test

A clinical dental researcher who has been involved in conservative dental treatment for several years created a knowledge test based on the fluoride topical application manual. The test consists of six items (Yes/No) related to oral care knowledge measuring oral complications (including oral infections and periodontal disease), physical signs of periodontal disease, and the consequences of periodontal disease and dry mouth (e.g., dental caries, dry mouth). The response options for these items were ‘true’, ‘false’ and ‘don’t know’. To confirm the reliability, the validity of the questionnaire was completed by six graduate Japanese students majoring in nursing, and Cronbach alpha value was 0.799.

### Motivation

#### Oral health behaviour motivation scale

The Oral Health Behavior Motivation Scale *(18 items, 5-Point Likert Scale)* is a reliable and valid scale for measuring motivation for oral health behaviors. This scale was developed in a study of 1,679 young Japanese adults, and consists of six factors and 18 items. [17]. This scale has six factors with three items, each with a Cronbach’s alpha value for each factor: α = 0.769 for factor 1, de-motivation; α = 0.862 for factor 2, allocentric adjustment; α = 0.728 for factor 3, incorporative adjustment; α = 0.796 for factor 4, integrated adjustment; α = 0.796 for factor 5, external adjustment; α = . 845 and factor 6 intrinsic motivation was α = 0.799. The 18 items are scored on a 5-point Likert scale (1 = *strongly disagree*, 2 = *disagree a little*, 3 = *neither agree nor disagree*, 4 = *agree a little*, 5 *= strongly agree*). The higher the score is, the higher the motivation for oral health behaviour.

### Behavioural skills

#### Self-efficacy scale for oral care

The Self-Efficacy Scale for Oral Care *(11 items, 5-Point Likert Scale)* has been assessed among 632 Japanese university students and found to be both valid and reliable [18]. This scale has four factors with Cronbach’s alpha values α = 0.876 for factor 1, α = 0.807 for factor 2, α = 0.813 for factor 3 and α = 0.703 for factor 4. The responses follow a 5-point Likert scale from 1 (*not confident)* to 5 (*completely confident*) for each item. Each subject is expressed as the sum of the scores of the five items and ranges from 5 to 25, with a higher score showing higher self-efficacy.

### Health behaviours

#### Fluoride use checklist

The Fluoride Use Checklist (4 items, *Yes/No)* is considered to be effective according to the latest oral care evidence [19]. The following were assessed: (a) brushing teeth with fluoride toothpaste at least twice a day, (b) paying attention to the concentration of fluoride when choosing a toothpaste, (c) rinsing the mouth only once after brushing, and (d) not eating or drinking for two hours after brushing.

### Oral health quality of life

#### General oral health assessment index (GOHAI)

The GOHAI scale *(12 items, 5-point Likert scale)* is validated in an elderly Japanese population. It is a comprehensive measure of health-related QoL related to dental caries, with a published Cronbach’s alpha value of 0.89 [20]. The GOHAI consists of three domains (subscales) that measure the degree of limitation in physical and psychosocial aspects of life due to oral-related problems. An overall score of 12 items is used to evaluate the GOHAI, with 60 as the highest score indicating satisfactory oral health.

### Statistical analysis

All statistical analyses were performed using IBM SPSS Statistics 27 software.

Because this is a pilot study, statistical inference was not required. However, statistical tests were conducted in order to explore effect sizes and to provide information for the calculation of sample sizes needed to develop a full research study on the topic. Comparisons between the two groups were performed using the chi-squared test and t test. Only participants with all valid responses for both baseline and postintervention outcomes were included in each model.

The qualitative data from the individual open-ended questions were used to identify themes regarding the acceptability of the e-learning program to participants and how the study procedures and processes worked in practice.

### Ethical approval

This study was a pilot randomized controlled trial with two groups. The trial protocol was registered with UMIN-CTR (ID: UMIN000045883) on 27/10/2021. Ethical approval was obtained from St. Luke’s International University Ethics Review Committee (approval number: 20-A095). Informed written consent was obtained from all participants.

The study reports followed the CONSORT extension to pilot and feasibility trials guidelines. The data were collected from April 2021 to January 2022.

## Results

### Baseline characteristics

As a result of randomization, there were no significant differences in sociodemographic and oral health status between the two groups (Table 1).

**Table 1:** Comparison of Characteristics of the Intervention and Control Groups (N = 82)

	Intervention (n = 36)	Control (n = 46)
Variable	Mean (SD)	Mean (SD)
Age in years	29.3 (4.8)	29.28 (4.1)
	N (%)	N (%)
Gender		
Female	28 (77.8)	33 (71.7)
Education level		
Associate degree*	1 (2.8)	1 (2.2)
University degree	30 (83.3)	34 (73.9)
Master’s degree	3 (8.3)	4 (8.7)
University student	0	5 (10.9)
Others	2 (5.6)	2 (4.3)
Smokers	1 (2.8)	4 (8.7)
Medical professionals	4 (11.1)	5 (10.9)
Brushing frequency		
Once a day or less	1 (2.8)	1 (2.2)
2	13 (36.1)	22(47.8)
3	22 (61.1)	20 (43.5)
≥4	0	3 (6.5)
Number of teeth extracted due to dental caries		
0	26 (72.2)	38 (82.6)
1	3 (8.3)	1 (2.2)
2	0	2 (4.3)
≥3	5 (13.9)	4 (8.7)
Unknown	2 (5.6)	1 (2.2)
Filled tooth due to dental caries		
Yes	29 (80.6)	30 (65.2)
No	6 (16.7)	14 (30.4)
Unknown	1 (2.8)	2 (4.3)

* refer to a degree that can be earned by those who have graduated from a short-term, two-year college

### Feasibility of trial design

**Fig. 4:**
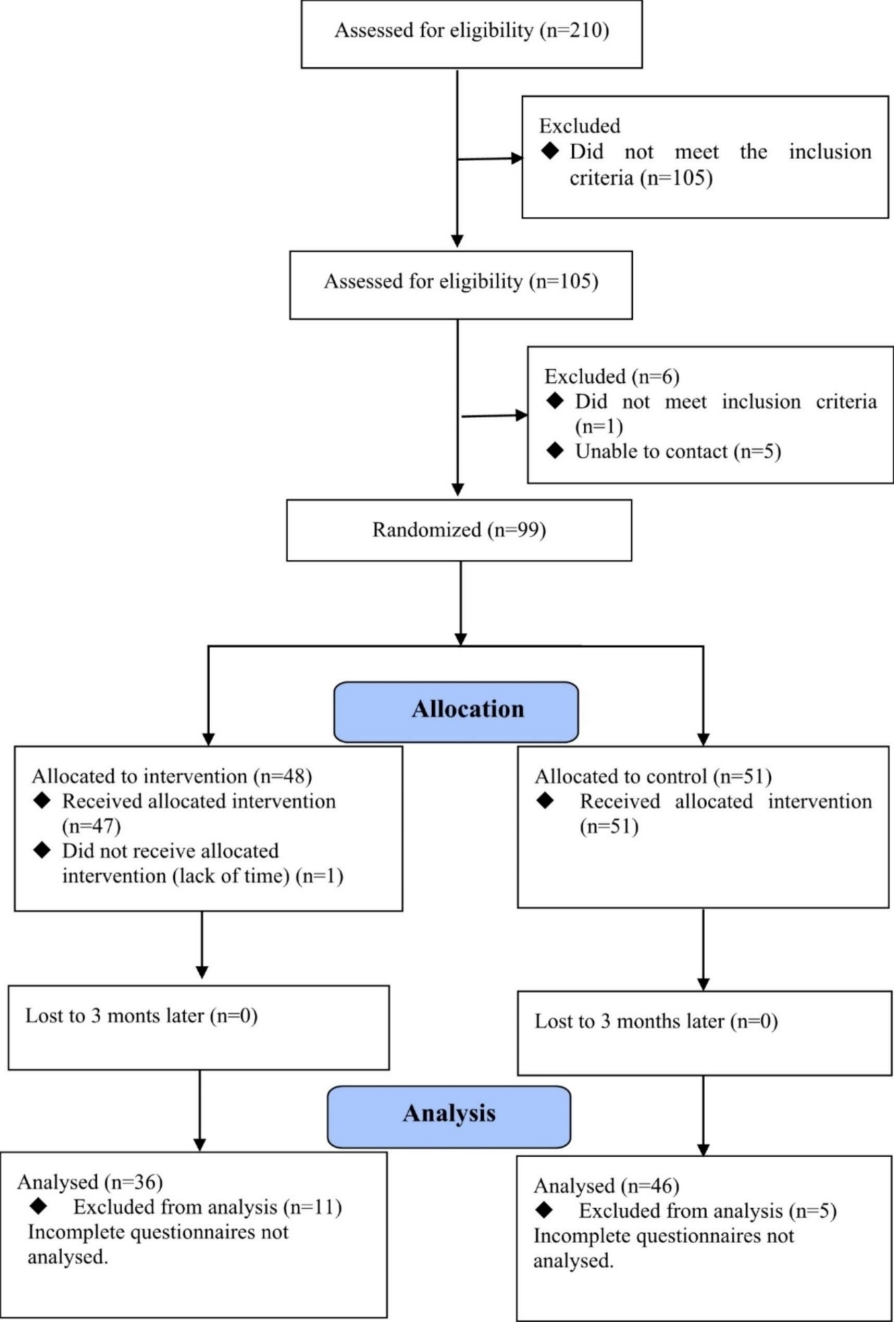
Trial Flow Diagram

Recruitment of participants began in April 2021, the final participants were randomized in October 2021, and the final follow-up outcome measures were completed in January 2022. (Fig. 4).

A total of 82 (82.8%) participants, 36 (75%) in the intervention group and 46 (90.2%) in the control group, who completed the post-test were included in the analysis. The dropout rate was 17.2%.

### Intervention acceptability

#### Time commitment and difficulty

The modal time taken to complete the e-learning was more than 30 min (33.3%). In the individual open-ended questions with three months in the intervention group, participants, 27 (75.0%) said that the e-learning program had changed their behaviour, 30 (83.3%) reported that the e-learning program was not difficult to understand, and 33 (91.7%) participants indicated that the e-learning materials have an impact on the promotion of oral health among overseas workers.

Feedback on the e-learning materials and program implementation was positive from all responding participants. Participants stated that they changed their tooth brushing behaviour after using the material and that they learned how to live a dental caries-free life.

#### Program quality and future content

Participants suggested improvements for the intervention; the most common was to use an easy-to-access medium and to make it available at any time. There was also a suggestion that the materials could be widely used by public organizations to disseminate information about oral health and include it in pretravel orientation, as in this study.

### Preliminary evidence of outcomes

#### Change in oral care knowledge, health behaviour motivation, self-efficacy scale for oral care, oral health-related QoL scores, and fluoride use behaviour

At the three months follow-up, oral care knowledge alone was improved in the e-learning group (Table 2). The changes in fluoride use behaviors in the intervention and control groups are shown in Table 3. At three months after the intervention, the intervention group showed a more positive impact than the control group with regard to “Paying attention to the concentration of fluoride when choosing a toothpaste” and “Not eating or drinking for two hours after brushing”.

**Table 2:** Changes in Outcome from Baseline to 3 Months Later by Status and Group

Variable	Intervention (36)				Control (46)				Difference Between Groups		
	Baseline		3 months		Baseline		3 months		3 months		
	Mean	SD	Mean	SD	Mean	SD	Mean	SD	MD	95% Cl	
Oral care knowledge	49.10	20.29	72.69	19.98	50.36	18.75	57.97	15.61	-14.71	-22.53 to -6.9	
Oral health behaviour motivation	51.81	8.74	55.94	9.51	53.20	9.07	54.0	8.57	-1.94	-5.93 to 2.04	
Self-efficacy scale for oral care	39.22	6.60	42.75	6.98	39.63	7.57	41.26	6.20	-1.49	-4.39 to 1.41	
Oral health-related QoL	48.69	8.04	49.86	8.06	47.96	7.58	48.00	6.94	-1.86	-5.16 to 1.44	

**Table 3:** Changes in Fluoride Use Behaviour from Baseline to 3 Months Later in the Intervention Group (n = 82)

	Baseline			3 months		
	**Intervention (36)**	**Control** **(46)**	**Intervention (36)**		**Control** **(46)**	**OR**
1. Brushing teeth with fluoride toothpaste at least twice a day. (YES)	20 (55.6)	27 (58.7)	28 (77.8)		38 (82.6)	0.74 (0.25 to 2.2)
2. Paying attention to the concentration of fluoride when choosing a toothpaste. (YES)	5 (13.9)	3 (6.5)	22 (61.1)		10 (21.7)	5.66 (2.15 to 14.92)
3. Rinsing mouth with water only once after brushing teeth. (Yes)	10 (27.8)	9 (19.6)	16 (44.4)		13 (28.3)	2.03 (0.81 to 5.09)
4. Not eating or drinking for two hours after brushing. (Yes)	11 (30.6)	8 (17.4)	22 (61.1)		12 (26.1)	4.45 (1.74 to 11.39)

The χ-square test was used to compare the control group with the intervention group

## Discussion

We evaluated the feasibility and acceptability of a theory-based self-care for oral health e-learning intervention in Japanese overseas workers measured at baseline and three months later in comparison with a control group. The study results showed that the theory-based e-learning program was a feasible and acceptable oral health behaviour intervention for expatriates in the target population.

### Feasibility

Regarding the feasibility of the study design, the high completion rate of the e-learning package and the positive evaluation of the e-learning learning materials suggest that although most research on oral care education has been conducted with children or dental students [21, 22], education for adults is also well tolerated.

One of the challenges we experienced was the small number of expatriates between the ages of 20 and 40 years who went abroad for long-term periods due to the spread of COVID-19. Since our program was focused on dental caries prevention at young and early middle stages, we excluded the middle-aged and older age groups. Therefore, in a full-scale RCT, recruitment during the recruitment period should be considered to increase the number of overseas workers who agree to participate in the study. For those who participated in the intervention, the rate of loss to follow-up was high, although few discontinued the intervention. As the study involved overseas workers, the posttest data collection was very time consuming. A longer posttest collection period from the outset, taking into account the time difference and internet availability when the questions were asked, would have reduced the drop-out rate.

According to the evaluations of the content, the illustrations and cartoons were highly appreciated. In the e-learning program in this study, the participants’ attention may have been attracted by the design and colours that were used to make the elements of the IMB model attractive to learn.

One of the negative comments was that it took too much time to learn the material. It takes approximately 40–45 min to watch the material. Considering that most participants spent 10–30 min watching the material, the content needs to be reduced slightly. For example, the information section had a lot of text and was content to educate dental students, so we could reduce it a bit with regard to content such as tooth erosion.

### Potential effect

Although this feasibility study had a limited sample size, it suggests the potential for the intervention to increase oral care knowledge. These results are not surprising, as IMB-based oral health messages promote changes in self-reported oral care knowledge [23]. However, because these are the results of a feasibility study with a limited sample size, the potential effect of the intervention should be confirmed in a RCT.

In a cluster randomized controlled study of children aged 10–11 years, the leaflet group showed higher oral health improvements than the e-learning group [21]. Internet-based health education is a new and unfamiliar approach for children. In some countries and locations, paper-based media such as leaflets will be more acceptable, children who do not have the knowledge to access websites may have difficult using their smartphones to access the entire website content. Evidence suggests that e-learning courses in oral health education can improve non-dental students’ knowledge and health behaviours in a study of dental and non-dental youth [24]. Although it is clear that oral health education can improve oral health knowledge attitudes and practice, teaching methods are often videos, instruction and group discussions, and there is not yet much research for adults on the use of web and e-learning [25, 26]. However, considering the results of our study and previous web and e-learning education results for young people, oral health e-learning programmes certainly have the potential to improve oral health behaviour.

Recent studies have shown that the use of high concentrations of fluoride is highly effective in preventing dental caries and periodontal disease [27]. In 2020, the WHO published a Model List of Essential Medicine describing the most effective, safe and cost-effective medicines for priority conditions. In this list, fluoride is shown as the minimum essential medicine for dental products, and a fluoride concentration of 1000 to 1500 ppm is recommended [28]. In the e-learning program we developed, the use of high-concentration fluoride was explained using the IMB model, and most participants in the intervention group who received e-learning indicated a potential improvement in their oral care knowledge. However, the effects of intervention materials need to be assessed with a sufficiently large sample size, as the interaction effects are not clear.

### Limitations

A potential limitation is that the results were based on self-reported data, and the use of more objective measures of data would help to improve the accuracy with which oral health behaviours are measured. In addition, because of the study setting, leaflets were also distributed to the e-learning group. It is possible that the oral health care effects of the leaflets may have influenced the results, but our difference-in-difference study design was intended to account for the shared effect of these leaflets in both groups, so this is unlikely to be a significant limitation. Another potential limitation is that the sample was recruited only from volunteer organizations in Japan; in addition, the study participants were mostly female, which may have affected the representativeness of the target population.

## Conclusions

This pilot study provides preliminary evidence of intervention success and evidence that the theory-based self-care for oral health e-learning intervention is feasible in overseas workers.

The study design was reasonably feasible; nevertheless, the content material requires shortening and slight modification. Future research should build on the results of this pilot study to test the effectiveness of the program in a larger and more diverse study group with longer follow-up periods. Such a study will help build improved oral health outcomes in overseas workers and enhance the workplace safety and wellbeing of an important segment of Japan’s overseas aid personnel.

## Data Availability

The dates used and analysed in this study are available from the corresponding author upon reasonable request.

## Abbreviations

IMB: information-motivation-behavioural skills
